# Exercise as a Disease-Modifying Therapy in Colon Cancer: Practice-Changing Evidence from the CHALLENGE Trial

**DOI:** 10.3390/cancers18172883

**Published:** 2026-09-06

**Authors:** Dai Shida

**Affiliations:** Division of Frontier Surgery, The Institute of Medical Science, The University of Tokyo, Tokyo 108-8639, Japan; dshida@g.ecc.u-tokyo.ac.jp; Tel.: +81-3-3443-8111; Fax: +81-3-5449-5604

**Keywords:** colon cancer, exercise oncology, physical activity, CHALLENGE trial, myokines, insulin/IGF-1 axis, disease-modifying therapy

## Abstract

Although robust observational data have long associated physical activity with improved colon cancer survival, the lack of randomized evidence has perpetuated a “causality gap,” confining exercise to supportive lifestyle advice and creating a clinical dilemma for surgeons. This paradigm has been fundamentally reshaped by the landmark phase III CHALLENGE trial. This review synthesizes the clinical, epidemiological, and translational evidence establishing exercise as a validated, disease-modifying intervention. In the CHALLENGE trial, a 3-year structured physical activity program significantly improved both disease-free survival (HR 0.72) and overall survival (HR 0.63) in patients with resected stage III or high-risk stage II colon cancer post-adjuvant chemotherapy, demonstrating a clinically meaningful additive benefit following completion of standard adjuvant chemotherapy. Mechanistically, exercise drives a multi-layered host–tumor reprogramming. Each acute exercise bout triggers transient surges of muscle-derived myokines (e.g., IL-6, SPARC) and immune-cell mobilization, enhancing natural killer and CD8^+^ T cell tumor infiltration. Cumulatively, these repeated acute pulses drive chronic adaptations, including downregulation of the pro-proliferative insulin/IGF-1 axis, attenuation of pro-tumor systemic inflammation, and microenvironmental remodeling at metastatic sites. To bridge the implementation gap, exercise oncology must transition from vague recommendations to structured, biomarker-driven clinical prescriptions defining type, dose, and duration. Mirroring the institutional success of Enhanced Recovery After Surgery (ERAS) protocols, physical activity should be systematically embedded into multidisciplinary, post-adjuvant oncologic care pathways. As the primary coordinators of colorectal cancer care, gastrointestinal surgeons must lead the transformation of exercise from optional supportive care into a validated, disease-modifying standard of care.

## Simple Summary

Observational studies have consistently linked physical activity to improved survival in colon cancer; however, a persistent “causality gap” has long prevented exercise from becoming standard oncological care. This review examines the paradigm shift brought by the international phase III CHALLENGE trial, which provides definitive causal evidence for a structured exercise program in patients with resected stage II/III colon cancer post-adjuvant chemotherapy. We synthesize the foundational epidemiological data, discuss the practice-changing outcomes of the trial, and elucidate the underlying molecular mechanisms—including downregulation of the insulin/IGF-1 axis and secretion of muscle-derived antitumor myokines. Finally, we address the practicalities of clinical implementation, providing a framework for transforming these data into actionable exercise prescriptions. Ultimately, this review shifts the clinical perspective of exercise from mere supportive care to an integral, disease-modifying component of standard colon cancer treatment.

## 1. Introduction

Despite significant advances in surgical techniques and perioperative care, colorectal cancer (CRC) remains a leading cause of global cancer mortality. While adjuvant chemotherapy has improved survival in Stage III colon cancer [1], the persistent risk of recurrence highlights a critical need for complementary strategies that transcend traditional tumor-directed treatments.

For decades, observational data have consistently linked post-diagnosis physical activity with improved disease-free survival (DFS) and overall survival (OS), often demonstrating a robust dose–response relationship [2]. However, these findings were long hindered by a “causality gap” due to inherent methodological limitations like confounding and reverse causality. Consequently, exercise was traditionally relegated to supportive care rather than recognized as a formal therapeutic intervention.

This long-standing paradigm has been fundamentally shifted by the landmark Colon Health And Life-Long Exercise ChaNGE (CHALLENGE) trial. As the first Phase III randomized controlled trial (RCT) in this setting, CHALLENGE demonstrated that a structured three-year exercise program significantly improves DFS and OS in patients with resected stage III or high-risk stage II colon cancer [3]. Importantly, exercise was evaluated as an add-on intervention following completed adjuvant chemotherapy rather than a substitute. This additive effect establishes structured exercise as a complementary disease-modifying strategy.

Reflecting this shift, 2026 clinical guidelines have been updated to reflect this new level of evidence. The European Society for Medical Oncology (ESMO) now recommends structured exercise as a core component of care [4], while the National Comprehensive Cancer Network (NCCN) assigns a Category 1 recommendation to moderate-intensity activity (≥150 min/week) for survivors [5]. These updates underscore the rapid transition of exercise from a general lifestyle suggestion to an evidence-based standard of care.

This review synthesizes the evolving evidence linking physical activity to CRC outcomes, focusing on the practice-changing implications of the CHALLENGE trial. We explore the epidemiological foundations, the biological mechanisms driving its antitumor effects, and practical strategies for clinical implementation, aiming to integrate exercise into the core of modern, precision-oriented oncologic care.

## 2. Pre-CHALLENGE Evidence: The Epidemiological Foundations and the Persistent “Causality Gap”

### 2.1. Strength of Association: Consistency, Magnitude, and Dose–Response

Prior to the CHALLENGE trial, the hypothesis that exercise could modify the disease course of CRC was supported almost exclusively by observational evidence (see Table 1). Over two decades, prospective cohorts consistently showed a robust inverse association between post-diagnosis physical activity and mortality. A landmark meta-analysis of 18 studies (n = 31,873) by Qiu et al. (2020) reported a 37% reduction in all-cause mortality (HR 0.63; 95% CI 0.54–0.74) and a 36% reduction in CRC-specific mortality (HR 0.64; 95% CI 0.47–0.88) when comparing the highest versus lowest activity cohorts [2]. Notably, a clear dose–response relationship emerged: each 10 MET-h/week increase (roughly 3 h of brisk walking) yielded a 21–24% risk reduction. These findings were sufficiently compelling to be cited by the American Cancer Society (ACS) as primary evidence supporting physical activity recommendations for CRC survivors [6]. Subsequent large-scale syntheses, such as the ACSM Roundtable Report, confirmed these findings, reinforcing the narrative of a robust, dosage-dependent benefit [7]. These meta-analyses effectively proved the *association*, but not *causation*.

### 2.2. Granularity and Real-World Impact: Insights from Stage III Cohorts

Nested cohort studies within RCTs provided clinical granularity, particularly for high-risk Stage III disease. Analysis of the CALGB/SWOG 80702 cohort (n = 1696) revealed that patients achieving ≥18.0 MET-h/week of activity enjoyed a 10.6% absolute increase in 3-year DFS (87.1% vs. 76.5% for <3.0 MET-h/wk; *p* < 0.001) [8]. Crucially, even light-to-moderate activity (≥1.5 h/week) conferred benefits, offering a realistic postoperative goal.

Furthermore, Brown et al. [9] pooled CALGB 89803 and 80702 cohorts (n = 2876), demonstrating that highly active patients (≥18.0 MET-h/week) nearly eliminated the survival gap compared to the general population [9]. Remarkably, 3-year recurrence-free survivors who were highly active even surpassed the general population’s survival by 2.9 percentage points. This suggested that exercise might not just aid recovery but potentially “normalize” the excess mortality associated with a CRC diagnosis.

**Table 1:** Pivotal Pre-CHALLENGE Evidence Supporting Physical Activity as a Disease-Modifying Agent in Colorectal Cancer.

Study (Year) & Ref.	Study Design & Target Population	Sample Size (N)	Key Quantitative Findings	Clinical Relevance	Study Limitations
Qiu et al. (2020) [2]	Meta-analysis of 18 prospective cohort studies (CRC survivors)	31,873	Highest vs. lowest level of post-dx PA: All-cause mortality: HR 0.63 (95% CI: 0.54–0.74). CRC-specific mortality: HR 0.64 (95% CI: 0.47–0.88) Dose–response: Each 10 MET-h/wk increase → 21% lower all-cause and 24% lower CRC mortality.	Formed the primary evidence basis for the American Cancer Society (ACS) lifestyle guidelines. Solidified proof of a strong linear dose–response relationship.	Observational design subject to recall bias from self-reported physical activity questionnaires and potential unmeasured confounding.
Markozannes et al. (CUP Global) (2024) [10]	Systematic review and meta-analysis under WCRF Global Cancer Update Programme (16 observational studies)	82,220	Consistent inverse association between post-dx PA and CRC mortality/recurrence. Estimated overall risk reductions: 13% to 60% across diverse international cohorts.	Most authoritative pre-CHALLENGE systematic review establishing broad global evidence across diverse international cohorts.	Expert Panel graded evidence as ‘limited-suggestive’ due to potential residual confounding, selection bias, and reverse causality; explicitly highlighted need for phase III RCTs.
Brown et al. (CALGB 80702) (2023) [8]	Prospective cohort study nested within Phase III RCT of adjuvant chemotherapy (Stage III colon cancer)	1696	Highly active (≥18 MET-h/wk) vs. inactive (<3 MET-h/wk) patients: 3-year DFS: 87.1% vs. 76.5% (Absolute difference +10.6%; HR 0.68, 95% CI: 0.44–1.04). Similar improvements observed for OS and recurrence-free survival.	Minimized treatment selection bias by evaluating patients who all received standardized contemporary adjuvant chemotherapy (FOLFOX/CAPOX). Showed PA adds value onto standard systemic therapy.	Nested observational design within an RCT cohort; physical activity was self-reported and non-randomized, leaving open possibility of healthy user bias.
Brown et al. (CALGB pooled) (2025) [9]	Pooled analysis of CALGB 89803 and 80702 cohorts compared against an age-, sex-, and year-matched general population (MGP)	2876	Inactive patients (<3 MET-h/wk) had survival rates 10.8–17.1% lower than MGP. Highly active patients (≥18 MET-h/wk) nearly closed the survival gap. 3-year recurrence-free survivors with ≥18 MET-h/wk had 2.9% higher survival than MGP.	Demonstrated that high levels of physical activity can effectively normalize excess mortality associated with Stage III colon cancer, shifting survival metrics back toward general population baselines.	Relied on indirect comparison with general population matching cohorts; potential residual survival/immortal time bias in landmark analyses.
McTiernan et al. (ACSM) (2019) [7]	Systematic review and consensus statement synthesis of 6 meta-analyses (American College of Sports Medicine)	Multiple cohorts	Dose–response reduction in all-cause mortality per increment of post-dx PA: +5 MET-h/wk: 15% lower mortality +10 MET-h/wk: 28% lower mortality +15 MET-h/wk: 35% lower mortality	Established official ACSM exercise guidelines for cancer survivors. Solidified biological gradient (dose–response) concept in exercise oncology.	Synthesized observational data only; underscored the critical absence of randomized controlled trial (RCT) data for definitive clinical exercise prescription.

### 2.3. The Epidemiological Ceiling: Why Association Was Not Enough

Despite this overwhelming signal—confirmed by the World Cancer Research Fund’s Global Cancer Update Programme (CUP Global) [10] review of 82,220 patients—the evidence was graded only as “limited-suggestive” [10,11]. This cautious grading reflects not a weakness of the data, but the inherent ceiling of observational research.

Three interrelated biases underpin this limitation. First, confounding by indication remains unavoidable: patients who are healthier, more functional, and less burdened by comorbidities are inherently more likely to engage in physical activity. Second, reverse causation cannot be excluded; patients with subclinical disease recurrence may reduce activity levels before clinical detection, thereby exaggerating the apparent protective effect of exercise. Third, reliance on self-reported activity introduces measurement error and potential reporting bias. Together, these factors constitute the central “scientific elephant in the room”––whether physical activity truly improves survival, or simply identifies patients who were already more likely to have favorable outcomes.

### 2.4. The Surgeon’s Dilemma: Evidence Without Actionability

This “causality gap” created a clinical paradox. While the epidemiological signal was potent and biologically plausible, the lack of randomized evidence precluded its integration into standard treatment algorithms. Consequently, surgeons could *encourage* exercise as supportive care, but could not *prescribe* it as disease-modifying therapy.

### 2.5. The Unavoidable Next Step

Against this backdrop, the need for a definitive RCT became increasingly clear. The field had reached the limits of what observational epidemiology could provide. Bridging the causality gap was no longer a theoretical objective, but a clinical imperative. In this context, the CHALLENGE trial was not simply an incremental contribution—it was the necessary experiment to determine whether the consistent associations observed over decades could be translated into causal, actionable evidence.

## 3. The CHALLENGE Trial: A Practice-Changing Randomized Evaluation of Exercise in Colon Cancer

**Figure 1:**
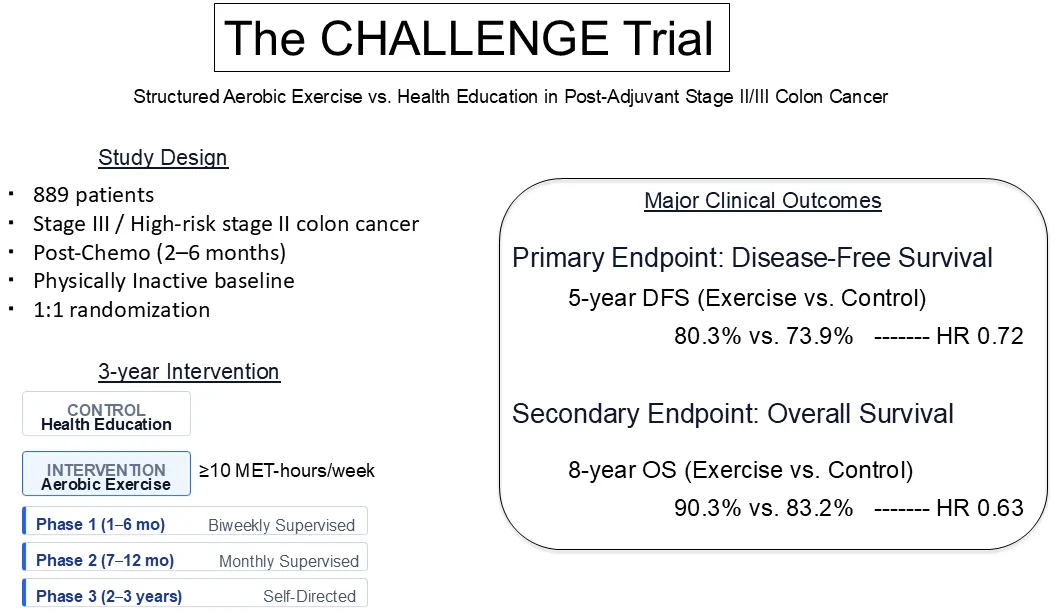
Summary of the CHALLENGE trial.

The Colon Health And Life-Long Exercise ChaNGE (CHALLENGE) trial represents a landmark phase III RCT providing the first definitive evidence that structured physical activity improves survival in colon cancer [3]. Conducted across 55 centers globally [3], this study bridged the long-standing gap between observational association and causal inference, establishing exercise as a disease-modifying intervention (Figure 1).

### 3.1. Study Design

The CHALLENGE trial enrolled 889 patients with resected (R0) stage III or high-risk stage II colon adenocarcinoma 2–6 months post-adjuvant chemotherapy. Participants were randomized (1:1) to either a structured exercise program or a health education control group. Eligibility required a physically inactive baseline (<150 min/week) and ECOG 0–1. The primary endpoint was DFS, with key secondary endpoints including OS and safety. Patients with rectal cancer were excluded from the trial to avoid confounding by distinct clinical and treatment variables—such as the use of neoadjuvant or adjuvant pelvic radiotherapy and surgical total mesorectal excision—and to ensure a homogeneous study population focused on colon adenocarcinoma [3,12]. Because the trial strictly required an ECOG performance status of 0–1, baseline physical inactivity (<150 min/week), and resected Stage II (high-risk) or Stage III colon cancer (excluding rectal cancer and Stage IV disease), these findings cannot be directly generalized to frail or elderly patients, individuals who are already physically active, patients with rectal cancer, or those with metastatic disease.

### 3.2. Intervention Framework: Exercise as a Behavioral Therapy

The program targeted an increase of ≥10 MET-h/week (approximately 150–300 min of moderate activity) above baseline. This behavioral intervention spanned three years across three phases: initiation (months 1–6; intensive supervision), consolidation (months 7–12), and maintenance (years 2–3). This design emphasizes that efficacy depends on sustained behavioral change rather than mere recommendation.

### 3.3. Efficacy: Evidence of Disease Modification

At a median follow-up of 7.9 years, the structured exercise program reduced the risk of disease recurrence, new primary cancer, or death by 28% (DFS: HR 0.72; 95% CI, 0.55–0.94), with a 6.4% absolute improvement in 5-year DFS (80.3% vs. 73.9%). Separation of the Kaplan–Meier curves emerged at approximately one year and persisted throughout follow-up, indicating a durable treatment effect. Importantly, this benefit translated into a significant OS advantage (OS: HR 0.63; 95% CI, 0.43–0.94). Reduced liver metastasis and second primary cancers, alongside similar non-cancer mortality between groups, support a direct effect of exercise on tumor biology.

### 3.4. Safety and Clinical Implications

Adverse events were predominantly low-grade musculoskeletal issues, with no treatment-related mortality. By demonstrating that structured exercise provides substantial post-chemotherapy risk reduction with minimal toxicity, the CHALLENGE trial elevates exercise from a prognostic lifestyle factor to a validated additive therapeutic strategy that should be integrated into standard post-adjuvant care.

## 4. Biological Mechanisms Underlying the Antitumor Effects of Exercise

**Figure 2:**
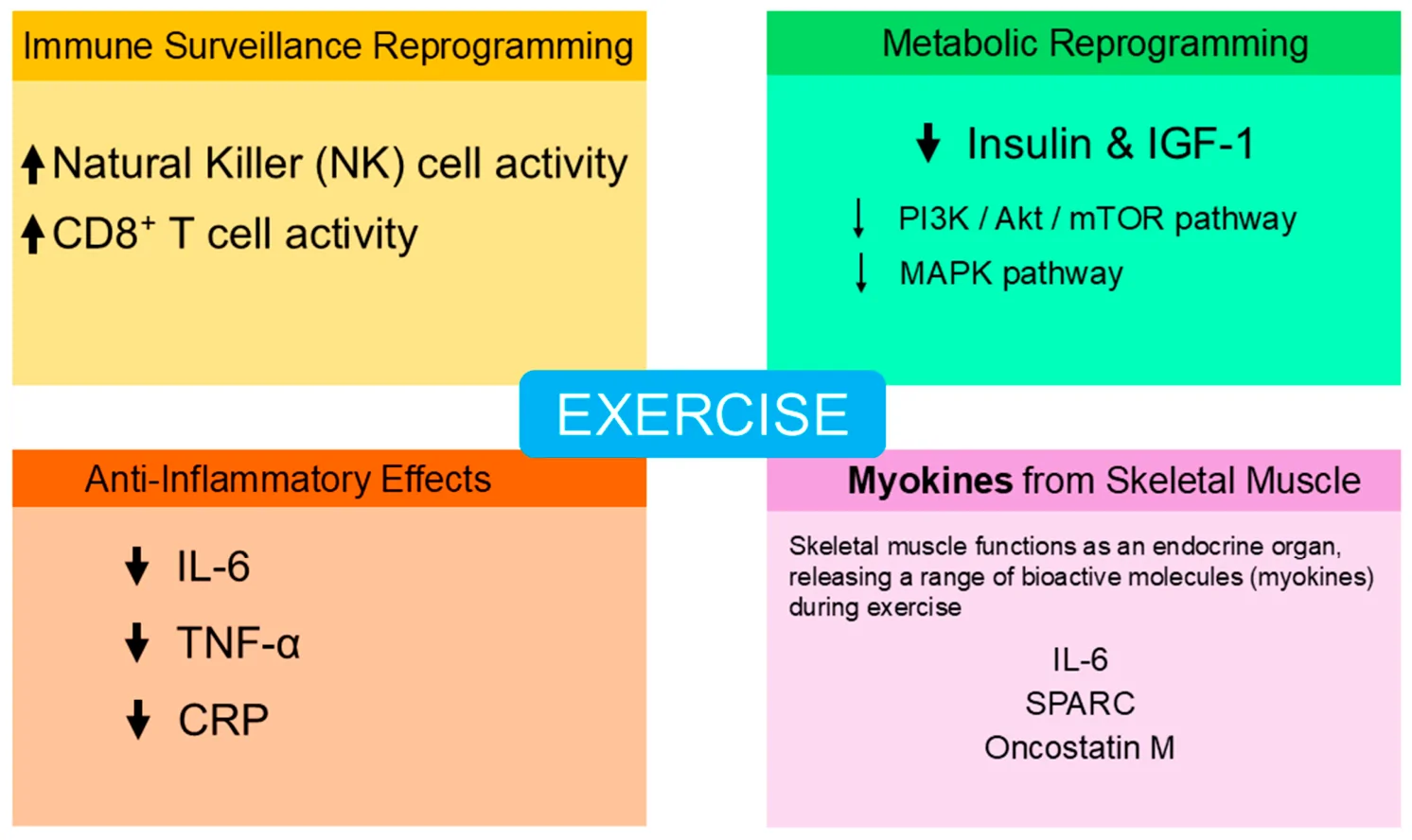
Biological mechanisms of exercise-induced antitumor effects. Exercise triggers a systemic reprogramming across four domains to reduce tumor progression and improve survival: Immune Surveillance Reprogramming: Increased activity of Natural Killer (NK) cells and CD8^+^ T cells. Metabolic Reprogramming: Downregulation of the Insulin/IGF-1 axis and inhibition of PI3K/Akt/mTOR and MAPK pathways. Anti-Inflammatory Effects: Reduction in chronic inflammatory mediators, including IL-6, TNF-α, and CRP. Myokines from Skeletal Muscle: Release of muscle-derived bioactive molecules (e.g., IL-6, IL-15, Oncostatin M, SPARC) that influence tumor biology.

The clinical efficacy demonstrated in the CHALLENGE trial is driven by complex, multifactorial biological mechanisms. Rather than acting through a single pathway, exercise induces a systemic reprogramming of host–tumor interactions across immune, metabolic, inflammatory, and myokines, creating a biological milieu hostile to tumor progression (Figure 2). It is important to emphasize that formal biomarker mediation analyses from the CHALLENGE trial itself have not yet been published. The underlying mechanisms discussed below are primarily synthesized from preclinical mouse models, in vitro studies, and observational human cohorts. While these pathways offer a plausible biological framework, attributing the survival benefits of the CHALLENGE trial directly to these specific pathways without formal mediation testing remains hypothetical and requires rigorous verification in trial-derived biospecimens.

### 4.1. Immune Surveillance Reprogramming: Coordinated Activation of NK Cells and CD8^+^ T Cells

Exercise induces rapid and coordinated activation of both innate and adaptive antitumor immunity, primarily through natural killer (NK) cells and CD8^+^ T cells. NK cells provide rapid, MHC-unrestricted killing via perforin/granzyme release and TRAIL-mediated apoptosis, while CD8^+^ T cells deliver antigen-specific cytotoxicity through TCR-MHC class I recognition [13,14]. Preclinical studies demonstrate that exercise-dependent tumor suppression is largely abrogated by NK-cell depletion [15] or β-adrenergic blockade [16]. Concurrently, exercise promotes CD8^+^ T-cell activation and tumor infiltration, with human data linking habitual physical activity to increased intratumoral CD8^+^ density in CRC [17].

Critical NK cell–CD8^+^ T cell crosstalk ensures effective antitumor immunity; depletion of either population impairs the other, while combined activation yields synergistic tumor control [18]. A key integrative mediator is interleukin-15 (IL-15), released from skeletal muscle, which supports the proliferation and survival of both subset [19]. Collectively, exercise fosters a systems-level enhancement of immune surveillance through this integrated effector network.

### 4.2. Metabolic Reprogramming: Modulation of the Insulin/IGF Axis

Exercise profoundly modulates the insulin/IGF axis, a primary driver of tumor growth and survival. Hyperinsulinemia and elevated IGF-1 signaling activate PI3K/Akt/mTOR and MAPK pathways, enhancing proliferation while inhibiting apoptosis [20,21]. Elevated insulin and C-peptide levels are linked to worse survival outcomes [22,23], while higher IGF-binding protein levels correlate with improved CRC prognosis [22].

By improving insulin sensitivity and reducing IGF-1 bioavailability, exercise attenuates these proliferative signals. Meta-analyses and trials in CRC populations confirm that exercise significantly reduces circulating IGF-1, often independently of weight loss [24,25,26].

In the CHALLENGE trial, improved oncologic outcomes without differences in non-cancer mortality suggest tumor-specific mechanisms, potentially mediated by this metabolic reprogramming [3]. Ongoing serial blood sample analyses from the trial will further clarify causal relationships between these biomarkers and clinical outcomes.

### 4.3. Anti-Inflammatory Effects: Attenuation of Tumor-Promoting Inflammation

Chronic systemic inflammation drives colon cancer progression;. elevated interleukin-6 (IL-6), tumor necrosis factor-alpha (TNF-α), and C-reactive protein (CRP) correlate with increased recurrence and mortality [27]. Regular exercise exerts potent anti-inflammatory effects by modulating these mediators.

Crucially, this operates via a paradoxical mechanism: acute exercise transiently increases muscle-derived IL-6, which triggers an anti-inflammatory cascade (e.g., increased IL-1 receptor antagonist (IL-1ra) and IL-10, suppressed TNF-α). Repeated “anti-inflammatory pulses” from regular training eventually lower baseline levels of CRP, TNF-α, and systemic IL-6 [28,29]. Randomized studies in cancer survivors demonstrate that structured exercise reduces circulating levels of CRP and IL-6 [30]. Given their strong prognostic impact in stage III colon cancer, this pathway represents a plausible candidate mechanism that warrants formal mediation testing in prospective trial cohorts.

### 4.4. Myokines: Skeletal Muscle as an Endocrine Organ

Skeletal muscle functions as an endocrine organ, releasing a range of bioactive molecules (myokines) during exercise that influence tumor biology. Key antitumor myokines include IL-6 [29,31], SPARC [32,33], and oncostatin M [34], with SPARC and IL-6 demonstrating the most robust direct efficacy against CRC. Notably, SPARC is the only myokine with a proven causal role in exercise-mediated CRC prevention; its protective effect against aberrant crypt foci is completely abolished in SPARC-deficient mice [32]. Similarly, IL-6 contributes causally, as recombinant IL-6 mimics the anti-proliferative and DNA-repair effects of exercise-conditioned serum on CRC cells [31].

The antitumor efficacy of myokines is driven by transient surges during acute exercise rather than chronic changes in resting levels. This highlights that frequent and consistent exercise is essential to maintain these cumulative clinical benefits [35,36,37].

### 4.5. Multi-Layered Reprogramming of Host–Tumor Interactions: Insights from the Exercise-Conditioned Serum Model

**Figure 3:**
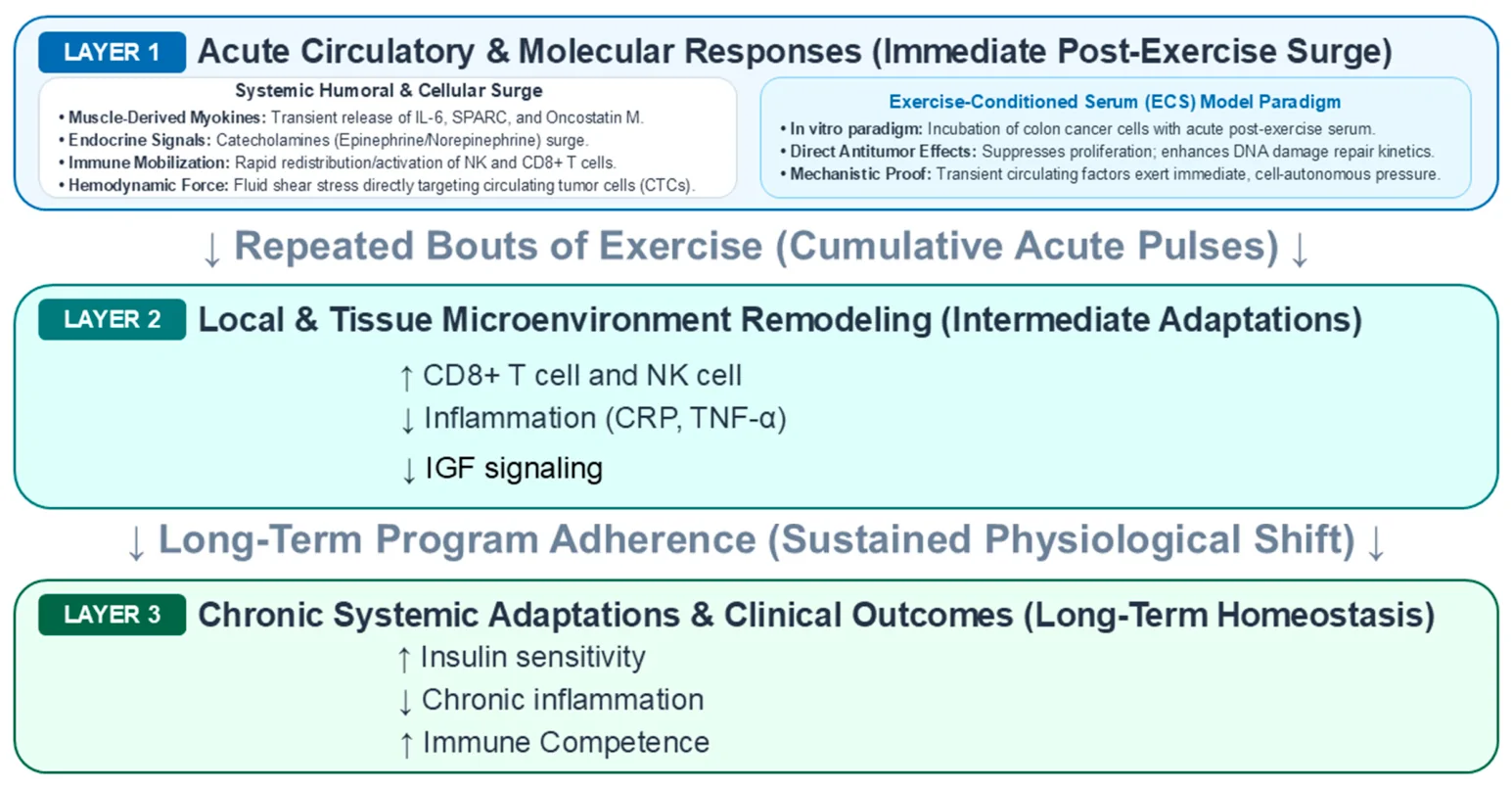
Multilayered Reprogramming of Antitumor Immunity and Clinical Outcomes. This model illustrates the hierarchical biological response to exercise in colon cancer across three layers: Layer 1 (Acute Reaction): A single bout triggers transient surges in circulating factors (e.g., myokines, catecholamines) and NK cells. The exercise-conditioned serum (ECS) model captures this immediate antitumor pressure. Layer 2 (Tissue Remodeling): Repeated bouts drive adaptations in the tumor microenvironment and metastatic sites, including reduced inflammation/IGF signaling and increased immune cell infiltration. Layer 3 (Systemic Adaptation): Chronic training establishes long-term metabolic and immune competence, including improved insulin sensitivity and reduced chronic inflammation. Together, these layers transform the host environment into one that is restrictive to tumor growth.

Exercise induces a coordinated, multi-layered reprogramming of the host environment, targeting cancer progression through systemic and site-specific mechanisms (Figure 3). Beyond metabolic and immunologic shifts, exercise exerts direct hemodynamic influences; for instance, the fluid shear stress generated during vigorous activity can mechanically destroy circulating tumor cells (CTCs), potentially reducing metastatic seeding [38]. Furthermore, exercise promotes vascular normalization and hypoxia reduction within the tumor microenvironment [39], while creating an “exercise-induced metabolic shield” in organs like the liver by limiting nutrient availability for tumor growth [40]. These effects are particularly critical for eradicating micrometastatic disease post-surgery.

The exercise-conditioned serum (ECS) model provides a robust framework for capturing these integrated effects. Serum obtained immediately post-exercise consistently suppresses cancer cell proliferation and enhances DNA damage repair (e.g., via polynucleotide kinase/phosphatase upregulation), primarily mediated by transient surges in myokines like IL-6, catecholamines, and metabolites [31,35,37,41]. While these effects are transient, diminishing within hours, they are reproducible with repeated bouts. This supports a “cumulative acute effect” model, where frequent, transient molecular surges collectively drive long-term clinical benefits.

Ultimately, these interconnected pathways—acute immune/factor release, intermediate tumor microenvironment remodeling, and chronic systemic adaptations—establish a host environment that is fundamentally less permissive to tumor progression. While these candidate pathways provide compelling hypotheses, definitive proof that they mediate the survival benefit observed in the CHALLENGE trial awaits published biomarker mediation analyses.

## 5. Clinical Implementation: Translating Evidence into Practice

The landmark CHALLENGE trial established structured exercise as an effective post-adjuvant, disease-modifying therapy in resected stage II/III colon cancer. Reflecting this evidence, updated clinical practice guidelines—including NCCN Survivorship Guidelines (Category 1 recommendation) [5] and ESMO updates—now place the operational focus on real-world execution. Consequently, the central question in surgical oncology has shifted from whether exercise provides oncologic benefit to how standardized referral pathways and precise exercise prescriptions can be systematically integrated into routine post-adjuvant care.

### 5.1. A Paradigm Shift in Surgical Oncology

The implications of CHALLENGE trial extend beyond survivorship care and directly reshape the role of the surgeon. Traditionally focused on achieving oncologic clearance, surgeons are now positioned to initiate interventions that influence long-term disease trajectory.

The postoperative period represents a critical “teachable moment,” during which patients are particularly receptive to behavioral change. Within this window, exercise should be introduced not as general lifestyle advice, but as a therapeutic strategy with defined oncologic benefit. Accordingly, exercise must be conceptualized alongside surgery, chemotherapy, and targeted therapy as a component of comprehensive cancer treatment. The key challenge is no longer to establish efficacy but to ensure consistent and structured delivery.

### 5.2. Exercise Prescription: What, How Much, and How Long?

Translating the CHALLENGE trial into practice requires moving from general recommendations to structured prescriptions.

What (Type): The CHALLENGE trial emphasized moderate-to-vigorous aerobic activity, most commonly brisk walking (about 4 METs), while allowing for patient preference [3]. Current guidelines (NCCN and ACSM guidelines) additionally recommend 2–3 sessions/week of resistance training targeting major muscle groups, plus stretching and balance exercises [5,42].

How Much (Dose): The target in the CHALLENGE trial was an increase of ≥10 MET-h/week above baseline, equivalent to approximately 45–60 min of brisk walking 3–4 times per week, or 25–30 min of jogging 3–4 times per week [3]. Current guidelines recommend a minimum of 150 min/week of moderate-intensity or 75 min/week of vigorous activity, with an ultimate goal of 300 min moderate or 150 min vigorous per week [5].

How Long (Duration of Program): The CHALLENGE intervention spanned 3 years in three phases: Phase 1 (months 1–6) included biweekly in-person behavioral support and supervised exercise sessions; Phase 2 (months 7–12) continued biweekly support sessions (in-person or remote); and Phase 3 (years 2–3) transitioned to monthly sessions [3]. Notably, survival curves began to separate at approximately 1 year and continued to diverge over 10 years of follow-up, underscoring the importance of sustained, long-term adherence [3]. Collectively, these data support a model of exercise as a long-term, titratable therapy that should be individualized based on functional status, comorbidities, and treatment context.

### 5.3. Standardization of Exercise as Oncology Care

To overcome institutional inconsistencies, exercise oncology must be embedded into standardized treatment pathways. While ERAS protocols have successfully standardized short-term perioperative pathways [43,44], long-term exercise oncology requires a fundamentally different implementation paradigm centered on sustained 3-year behavioral change. Unlike short-term surgical pathways, delivering 3 years of structured intervention—including 6 months of biweekly supervised sessions—presents substantial resource demands, financial costs, and real-world implementation barriers.

Real-world translation faces significant challenges regarding cost-effectiveness and patient attrition. In trial conditions, long-term compliance is reinforced by clinical trial infrastructure; however, in resource-constrained real-world health systems, funding supervised facilities and dedicated exercise specialists over multiple years is a major hurdle. Furthermore, participant drop-out and declining adherence over time remain critical issues. Hybrid models utilizing digital health platforms, community-based exercise groups, and risk-stratified supervision (e.g., transitioning low-risk, motivated patients early to home-based programs) must be formally evaluated to ensure cost-effectiveness and sustained long-term engagement.

### 5.4. Future Directions: Precision Exercise Oncology

While the CHALLENGE trial provides definitive evidence of efficacy, it also highlights variability in patient response, suggesting a need for precision approaches. Future research should aim to identify predictive biomarkers that define responsiveness to exercise, incorporating host factors (e.g., immune competence, metabolic phenotype, systemic inflammation), tumor characteristics (e.g., molecular subtype) [45], and treatment context. The biological mechanisms outlined earlier—immune activation, insulin/IGF axis modulation, and tumor microenvironment remodeling—offer a rational framework for such stratification. Ultimately, the field must evolve from uniform recommendations toward individualized exercise prescriptions that maximize therapeutic benefit while minimizing patient burden. Surgeons must now take the lead in ensuring that exercise is not merely advised, but prescribed and systematically integrated into routine postoperative oncologic care.

## 6. Conclusions

The level-1 evidence from the landmark CHALLENGE trial transitions exercise in stage II/III colon cancer from observational advice to a structured, post-chemotherapy protocol for physically inactive, fit patients (ECOG 0–1). While these findings establish structured exercise as a validated, disease-modifying post-adjuvant therapy, caution is required before generalizing these results to rectal cancer, stage IV disease, frail populations, or baseline-active individuals. Moving forward, the clinical mandate requires overcoming real-world implementation barriers—specifically patient attrition, financial costs, and healthcare resource constraints—while refining biomarker-driven personalization and evaluating tailored exercise strategies for broader, unstudied oncologic cohorts. The remaining challenge is not further proof of efficacy, but the consistent and equitable integration of structured exercise into routine clinical care. Bridging this implementation gap will require coordinated efforts across disciplines, alongside continued advances in personalization and delivery. Ultimately, integrating physical activity into standard post-adjuvant oncologic care requires treating exercise with the same clinical rigor as pharmacological therapeutics—defining precise dosing, duration, and delivery pathways to optimize long-term outcomes for patients with colon cancer.

## Data Availability

No new data were created or analyzed in this study. Data sharing is not applicable to this article.

## Abbreviations

The following abbreviations are used in this manuscript:

CHALLENGE trial	The Colon Health And Life-Long Exercise ChaNGE trial
CRP	C-reactive protein
DFS	Disease-free survival
ECS	Exercise-conditioned serum
ESMO	European Society for Medical Oncology
MET	Metabolic equivalent task
NCCN	National Comprehensive Cancer Network
NK cells	Natural killer cells
OS	Overall survival

## References

[1] Andre T., Meyerhardt J., Iveson T., Sobrero A., Yoshino T., Souglakos I., Grothey A., Niedzwiecki D., Saunders M., Labianca R. (2020). Effect of duration of adjuvant chemotherapy for patients with stage III colon cancer (IDEA collaboration): Final results from a prospective, pooled analysis of six randomised, phase 3 trials. Lancet Oncol..

[2] Qiu S., Jiang C., Zhou L. (2020). Physical activity and mortality in patients with colorectal cancer: A meta-analysis of prospective cohort studies. Eur. J. Cancer Prev..

[3] Courneya K.S., Vardy J.L., O’Callaghan C.J., Gill S., Friedenreich C.M., Wong R.K.S., Dhillon H.M., Coyle V., Chua N.S., Jonker D.J. (2025). Structured Exercise after Adjuvant Chemotherapy for Colon Cancer. N. Engl. J. Med..

[4] Pentheroudakis G., Argiles G., Arnold D., Smyth E., Ducreux M. (2026). ESMO Clinical Practice Guideline Express Update on the adoption of physical exercise in patients with localised colon cancer. ESMO Open.

[5] National Comprehensive Cancer Network NCCN Clinical Practice Guidelines in Oncology (NCCN Guidelines^®^): Survivorship. https://www.nccn.org/guidelines/guidelines-detail?category=1&id=1461.

[6] Rock C.L., Thomson C.A., Sullivan K.R., Howe C.L., Kushi L.H., Caan B.J., Neuhouser M.L., Bandera E.V., Wang Y., Robien K. (2022). American Cancer Society nutrition and physical activity guideline for cancer survivors. CA Cancer J. Clin..

[7] McTiernan A., Friedenreich C.M., Katzmarzyk P.T., Powell K.E., Macko R., Buchner D., Pescatello L.S., Bloodgood B., Tennant B., Vaux-Bjerke A. (2019). Physical Activity in Cancer Prevention and Survival: A Systematic Review. Med. Sci. Sports Exerc..

[8] Brown J.C., Ma C., Shi Q., Fuchs C.S., Meyer J., Niedzwiecki D., Zemla T., Couture F., Kuebler P., Kumar P. (2023). Physical Activity in Stage III Colon Cancer: CALGB/SWOG 80702 (Alliance). J. Clin. Oncol..

[9] Brown J.C., Ma C., Shi Q., Saltz L.B., Shields A.F., Meyerhardt J.A. (2025). The association of physical activity with survival in colon cancer versus a matched general population: Data from Cancer and Leukemia Group B 89803 and 80702 (Alliance). Cancer.

[10] Markozannes G., Becerra-Tomas N., Cariolou M., Balducci K., Vieira R., Kiss S., Aune D., Greenwood D.C., Gunter M.J., Copson E. (2024). Post-diagnosis physical activity and sedentary behaviour and colorectal cancer prognosis: A Global Cancer Update Programme (CUP Global) systematic literature review and meta-analysis. Int. J. Cancer.

[11] Tsilidis K.K., Markozannes G., Becerra-Tomas N., Cariolou M., Balducci K., Vieira R., Kiss S., Aune D., Greenwood D.C., Dossus L. (2024). Post-diagnosis adiposity, physical activity, sedentary behaviour, dietary factors, supplement use and colorectal cancer prognosis: Global Cancer Update Programme (CUP Global) summary of evidence grading. Int. J. Cancer.

[12] Courneya K.S., Booth C.M., Gill S., O’Brien P., Vardy J., Friedenreich C.M., Au H.J., Brundage M.D., Tu D., Dhillon H. (2008). The Colon Health and Life-Long Exercise Change trial: A randomized trial of the National Cancer Institute of Canada Clinical Trials Group. Curr. Oncol..

[13] Liu L., Deng Z., Fang R., Zou M., Ren J., Gao Y., Peng J., Hao L. (2026). Exercise and CD8(+) T cells: Mechanisms of immune modulation in antitumor responses. J. Mol. Med..

[14] Fiuza-Luces C., Valenzuela P.L., Galvez B.G., Ramirez M., Lopez-Soto A., Simpson R.J., Lucia A. (2024). The effect of physical exercise on anticancer immunity. Nat. Rev. Immunol..

[15] Nagao F., Suzui M., Takeda K., Yagita H., Okumura K. (2000). Mobilization of NK cells by exercise: Downmodulation of adhesion molecules on NK cells by catecholamines. Am. J. Physiol. Regul. Integr. Comp. Physiol..

[16] Dimitrov S., Lange T., Born J. (2010). Selective mobilization of cytotoxic leukocytes by epinephrine. J. Immunol..

[17] Renman D., Gylling B., Vidman L., Boden S., Strigard K., Palmqvist R., Harlid S., Gunnarsson U., van Guelpen B. (2021). Density of CD3(+) and CD8(+) Cells in the Microenvironment of Colorectal Cancer according to Prediagnostic Physical Activity. Cancer Epidemiol. Biomark. Prev..

[18] Lanickova T., Angelidou A., Hensler M., Vankerckhoven A., Niederlova V., Vosahlikova S., Kasikova L., Bock A., Zacharova E., Stekas C. (2026). NKG2A inhibition promotes NK cell-CD8(+) T cell interactions to improve anticancer immunity in ovarian carcinoma. Nat. Commun..

[19] Kurz E., Hirsch C.A., Dalton T., Shadaloey S.A., Khodadadi-Jamayran A., Miller G., Pareek S., Rajaei H., Mohindroo C., Baydogan S. (2022). Exercise-induced engagement of the IL-15/IL-15Ralpha axis promotes anti-tumor immunity in pancreatic cancer. Cancer Cell.

[20] Sandhu M.S., Dunger D.B., Giovannucci E.L. (2002). Insulin, insulin-like growth factor-I (IGF-I), IGF binding proteins, their biologic interactions, and colorectal cancer. J. Natl. Cancer Inst..

[21] Hopkins B.D., Goncalves M.D., Cantley L.C. (2020). Insulin-PI3K signalling: An evolutionarily insulated metabolic driver of cancer. Nat. Rev. Endocrinol..

[22] Wolpin B.M., Meyerhardt J.A., Chan A.T., Ng K., Chan J.A., Wu K., Pollak M.N., Giovannucci E.L., Fuchs C.S. (2009). Insulin, the insulin-like growth factor axis, and mortality in patients with nonmetastatic colorectal cancer. J. Clin. Oncol..

[23] Gunter M.J., Hoover D.R., Yu H., Wassertheil-Smoller S., Rohan T.E., Manson J.E., Howard B.V., Wylie-Rosett J., Anderson G.L., Ho G.Y. (2008). Insulin, insulin-like growth factor-I, endogenous estradiol, and risk of colorectal cancer in postmenopausal women. Cancer Res..

[24] Haydon A.M., Macinnis R.J., English D.R., Giles G.G. (2006). Effect of physical activity and body size on survival after diagnosis with colorectal cancer. Gut.

[25] Kwon Y.R., Kim Y., Kim Y. (2025). Exercise-induced modulation of IGF-1 in healthy, obese, and cancer populations: A systematic review and meta-analysis. Ann. Med..

[26] Lee M.K., Kim J.Y., Kim D.I., Kang D.W., Park J.H., Ahn K.Y., Yang H., Lee D.H., Roh Y.H., Lee J.W. (2017). Effect of home-based exercise intervention on fasting insulin and Adipocytokines in colorectal cancer survivors: A randomized controlled trial. Metabolism.

[27] Cheng E., Shi Q., Shields A.F., Nixon A.B., Shergill A.P., Ma C., Guthrie K.A., Couture F., Kuebler P., Kumar P. (2023). Association of Inflammatory Biomarkers with Survival Among Patients with Stage III Colon Cancer. JAMA Oncol..

[28] Poorhabibi H., Weiss K., Rosemann T., Knechtle B., Eslami R., Tartibian B., Tayebi S.M., Sheikhhoseini R. (2025). Short-Lived Exercise-Induced Exerkines Modulate Inflammation for Chronic Disease Prevention: A Systematic Review and Meta-Analysis. Biomolecules.

[29] Orange S.T., Leslie J., Ross M., Mann D.A., Wackerhage H. (2023). The exercise IL-6 enigma in cancer. Trends Endocrinol. Metab..

[30] Brown J.C., Zhang S., Ligibel J.A., Irwin M.L., Jones L.W., Campbell N., Pollak M.N., Sorrentino A., Cartmel B., Harrigan M. (2020). Effect of Exercise or Metformin on Biomarkers of Inflammation in Breast and Colorectal Cancer: A Randomized Trial. Cancer Prev. Res..

[31] Orange S.T., Jordan A.R., Odell A., Kavanagh O., Hicks K.M., Eaglen T., Todryk S., Saxton J.M. (2022). Acute aerobic exercise-conditioned serum reduces colon cancer cell proliferation in vitro through interleukin-6-induced regulation of DNA damage. Int. J. Cancer.

[32] Aoi W., Naito Y., Takagi T., Tanimura Y., Takanami Y., Kawai Y., Sakuma K., Hang L.P., Mizushima K., Hirai Y. (2013). A novel myokine, secreted protein acidic and rich in cysteine (SPARC), suppresses colon tumorigenesis via regular exercise. Gut.

[33] Akutsu T., Ito E., Narita M., Ohdaira H., Suzuki Y., Urashima M. (2020). Effect of Serum SPARC Levels on Survival in Patients with Digestive Tract Cancer: A Post Hoc Analysis of the AMATERASU Randomized Clinical Trial. Cancers.

[34] Negrini K.A., Lin D., Shah D., Wu H., Wehrung K.M., Thompson H.J., Whitcomb T., Sturgeon K.M. (2024). Role of Oncostatin M in Exercise-Induced Breast Cancer Prevention. Cancers.

[35] Devin J.L., Hill M.M., Mourtzakis M., Quadrilatero J., Jenkins D.G., Skinner T.L. (2019). Acute high intensity interval exercise reduces colon cancer cell growth. J. Physiol..

[36] Farley M.J., Boytar A.N., Adlard K.N., Salisbury C.E., Hart N.H., Schaumberg M.A., Jenkins D.G., Skinner T.L. (2024). Interleukin-15 and high-intensity exercise: Relationship with inflammation, body composition and fitness in cancer survivors. J. Physiol..

[37] Bettariga F., Taaffe D.R., Borsati A., Avancini A., Pilotto S., Lazzarini S.G., Galvao D.A., Newton R.U. (2026). Acute and chronic exercise-conditioned serum effects on cancer cells in vitro: A systematic review. Eur. J. Cancer Prev..

[38] Regmi S., Fu A., Luo K.Q. (2017). High Shear Stresses under Exercise Condition Destroy Circulating Tumor Cells in a Microfluidic System. Sci. Rep..

[39] Betof A.S., Lascola C.D., Weitzel D., Landon C., Scarbrough P.M., Devi G.R., Palmer G., Jones L.W., Dewhirst M.W. (2015). Modulation of murine breast tumor vascularity, hypoxia and chemotherapeutic response by exercise. J. Natl. Cancer Inst..

[40] Sheinboim D., Parikh S., Manich P., Markus I., Dahan S., Parikh R., Stubbs E., Cohen G., Zemser-Werner V., Bell R.E. (2022). An Exercise-Induced Metabolic Shield in Distant Organs Blocks Cancer Progression and Metastatic Dissemination. Cancer Res..

[41] Metcalfe R.S., Kemp R., Heffernan S.M., Churm R., Chen Y.C., Ruffino J.S., Conway G.E., Tornillo G., Orange S.T. (2021). Anti-carcinogenic effects of exercise-conditioned human serum: Evidence, relevance and opportunities. Eur. J. Appl. Physiol..

[42] Campbell K.L., Winters-Stone K.M., Wiskemann J., May A.M., Schwartz A.L., Courneya K.S., Zucker D.S., Matthews C.E., Ligibel J.A., Gerber L.H. (2019). Exercise Guidelines for Cancer Survivors: Consensus Statement from International Multidisciplinary Roundtable. Med. Sci. Sports Exerc..

[43] Fearon K.C., Ljungqvist O., Von Meyenfeldt M., Revhaug A., Dejong C.H., Lassen K., Nygren J., Hausel J., Soop M., Andersen J. (2005). Enhanced recovery after surgery: A consensus review of clinical care for patients undergoing colonic resection. Clin. Nutr..

[44] Shida D., Tagawa K., Inada K., Nasu K., Seyama Y., Maeshiro T., Miyamoto S., Inoue S., Umekita N. (2015). Enhanced recovery after surgery (ERAS) protocols for colorectal cancer in Japan. BMC Surg..

[45] Shida D., Kuchiba A., Shibata T., Hamaguchi T., Yamasaki S., Ito M., Kobatake T., Tonooka T., Masaki T., Shiozawa M. (2023). Genomic landscape and its prognostic significance in stage III colorectal cancer: JCOG1506A1, an ancillary of JCOG0910. Cancer Sci..

